# Mitochondrial Dysfunction Across Multiple Organs in Broilers Exhibiting Wooden Breast Myopathy

**DOI:** 10.3390/ani16203285

**Published:** 2026-10-18

**Authors:** Majid Shakeri, Debolina Chatterjee, Hamid Reza Rafieian-Naeini, Woo Kyun Kim, Byungwhi Kong, Brian Bowker

**Affiliations:** 1 Oak Ridge Institute for Science and Education, Oak Ridge, TN 37830, USA; 2 USDA-ARS, U.S. National Poultry Research Center, Athens, GA 30605, USA; debolina.chatterjee@usda.gov (D.C.); byungwhi.kong@usda.gov (B.K.); 3 Department of Poultry Science, University of Georgia, Athens, GA 30602, USA; hamidreza.rafieiannaeini@uga.edu (H.R.R.-N.); wkkim@uga.edu (W.K.K.)

**Keywords:** broiler, breast myopathy, meat quality, mitochondrial function, wooden breast

## Abstract

Wooden breast (WB) in broilers has been linked to impaired mitochondrial function in the breast muscle. When mitochondrial function declines, organs receive less ATP and experience oxidative stress. Muscle development is linked to the optimal function of the gut, liver, and pancreas, which depends on adequate ATP to support nutrient absorption and metabolism. The effects of the WB myopathy on other organs and tissues within broilers are relatively unknown. The aim of this study was to investigate factors linked to mitochondrial function in multiple organs from broilers exhibiting WB. From sixty 8-week-old broilers, ten severe WB and ten normal breast muscles were selected for tissue collection (liver, pancreas, three sections of intestine, and breast muscle). Reduced citrate synthase activity and mitochondrial complex-II activity were observed in the muscle, pancreas, liver and duodenum tissues of WB broilers compared to normal broilers. Broilers with WB had reduced glutathione content in the pancreas and all three sections of the intestine when compared to normal broilers. Whole tissue ATP content was decreased in the muscle and liver of WB broilers. Overall, these data suggest that mitochondrial function may be impaired in tissues beyond just the breast muscle in broilers that exhibit the WB myopathy.

## Simple Summary

Broilers with the wooden breast (WB) myopathy showed mitochondrial dysfunction beyond the breast muscle. Broilers exhibiting WB had reduced citrate synthase activity, glutathione content, mitochondrial complex-II activity, and ATP levels across multiple organs when compared to normal broilers (no WB). The results of this study suggest systemic impairment in energy production across multiple organs. Furthermore, the decline in mitochondrial function across multiple organs indicates that the development of WB in the breast muscle could be linked to broader metabolic disruptions throughout the body of the broilers and merits further investigation. Overall, broilers with WB exhibited less functional mitochondria and greater oxidative stress in multiple tissues.

## 1. Introduction

The wooden breast (WB) condition is a myopathy that occurs in the *Pectoralis major* of broiler chickens that leads to abnormal breast meat texture and poor meat quality. Although the condition poses no health risks to consumers, the WB myopathy has a negative economic impact on the poultry industry due to product downgrades [1]. The specific etiology of WB is currently unknown, and there is no definitive solution to the problem. As the WB myopathy manifests itself in the *Pectoralis major*, most research on this topic has focused solely on this muscle, and relatively little is known about how other tissues and organs are impacted in broilers exhibiting this condition. Previous reports have shown some gene expression and biochemical differences in liver, duodenum, and pancreas tissues from broilers exhibiting the WB myopathy compared to normal broilers [2]. Biomedical research suggests a systemic interaction between skeletal muscle homeostasis and metabolic organs such as the liver, pancreas, and gut [3]. A healthy digestive system comprising a functional liver, pancreas, and balanced gut microbiota is important for optimal nutrient absorption, directly limiting intramuscular lipid oxidation and excessive abdominal fat deposition [4,5].

Among the many factors that can affect organ health, mitochondria are particularly important, as they serve as a major source of ATP production and contribute to the removal of excess reactive oxygen species (ROS) from the body. Mitochondria are double-membrane organelles found in the cytoplasm of most eukaryotic cells. Their main function is to produce most of the ATP required by cells through aerobic respiration. Mitochondrial function is closely linked to the citric acid cycle (TCA), which serves as the core metabolic engine of the organelle [6]. Among several factors, citrate synthase and mitochondrial complex-II are important parts of the TCA cycle. In addition to their role in energy metabolism, mitochondria are also important for cell signaling and cell death [7]. Dysfunctional mitochondria overproduce ROS, which can lower ATP production and ultimately lead to cell death and inflammation [8]. Several studies have reported mitochondrial abnormalities in the breast muscle of broilers exhibiting WB [9,10]. Available data suggest that mitochondrial abnormalities in the breast muscle of broilers may play a role in WB development by disturbing the conversion of ROS and ATP production, leading to greater oxidative damage to the tissues [11].

It was hypothesized that mitochondrial function in organs other than the breast muscle may be altered in broilers with the WB condition. The specific objective of this study was to identify mitochondrial-related differences between normal broilers (no WB) and those exhibiting severe WB across multiple organs and tissues, particularly those of the digestive system. Understanding the level of mitochondrial function across different organs involved in gut and muscle health may help identify potential strategies for addressing WB in broilers. To obtain tissue-specific data, mitochondria were isolated from the tissues (muscle, liver, pancreas, duodenum, ileum, and jejunum) of normal and WB broilers and evaluated. For this study, citrate synthase and mitochondrial complex-II activities were measured as biochemical indicators of mitochondrial function.

## 2. Materials and Methods

### 2.1. Animal Handling and Sampling

A total of sixty 8-week-old male Ross-308 broiler chickens that were raised on commercial starter (AME: 2975 kcal/kg and CP: 22.5), grower (AME: 3000 kcal/kg and CP: 20.5), and finisher (AME: 3100 kcal/kg and CP: 19.5) diets were randomly selected from a local research farm (University of Georgia, Athens, GA, USA). Broilers were fasted for 12 h before slaughtering. Broilers were weighed and slaughtered via electrical stunning and exsanguination at the U.S. National Poultry Research Center pilot processing facility (Athens, GA, USA). To ensure consistent post-mortem assessment and sampling times, broilers were slaughtered in batches of 2–3 birds at a time. Immediately following bleed out, the skin on the breast portion of the carcass was removed, and breast muscles (*Pectoralis major*) were assessed for the WB condition by trained experts using tactile palpation and visual assessment as described previously [12]. Carcasses were designated as having severe WB if both the left and right breast muscles exhibited characteristic abnormal hardness throughout the entire length of the muscles, the out-bulging shape on the cranial ends, and the ridge-like shape on the caudal ends of the muscles. Carcasses were classified as normal (no WB) if neither breast muscle exhibited any degree of WB characteristics. Ten carcasses exhibited severe WB and ten carcasses exhibited no degree of WB (normals) and were utilized for subsequent tissue sampling and evaluation. Carcasses that displayed only mild or moderate degrees of WB myopathy were not utilized in this study. From the selected carcasses, tissue samples from three sections of the intestine (duodenum, jejunum, and ileum), pancreas, liver, and breast muscle (*Pectoralis major*) were removed within 15 min post-mortem; immediately frozen in liquid nitrogen; and stored at −80 °C for further analysis. Tissue samples from the duodenum, jejunum, and ileum were collected from the middle portion of each respective section to ensure sampling location consistency across birds. Breast muscle samples were collected from the cranial–ventral portion (skin side) of the *Pectoralis major*. The breast muscle side that was not used for tissue sampling was deboned within 15 min post-mortem, reassessed to verify the WB condition, placed in a sealed plastic bag, and stored at 4 °C until 24 h post-mortem for meat quality measurements.

### 2.2. Meat Quality Measurements

Meat pH at 24 h post-mortem was measured in the cranial portion of each breast muscle using a spear-tipped probe and pH meter (Thermo Scientific, Pittsburgh, PA, USA). Compression force (N) was measured on the skin-side surface of the raw breast meat using a Texture Analyzer (TA.XTplus Texture Analyzer, Stable Micro System, Godalming, UK) equipped with an aluminum rounded-edge cylindrical probe (6 mm diameter) and a 50 kg load cell. The breast meat was compressed 6 mm at a 5 mm/sec test speed from the initial contact. Breast meat color values (L*, a* and b*) were measured at 24 h post-mortem on the ventral (skin-side) and dorsal (bone-side) surfaces of the muscles using a Minolta CM-700d spectrophotometer (Konica Minolta Inc., Ramsey, NJ, USA). Drip loss percentage was calculated based on the difference between initial muscle weight (initial 15 min post-mortem) and muscle weight after 12 and 24 h of storage at 4 °C.

### 2.3. Mitochondrial Extraction and Biochemical Assays

Mitochondrial-enriched fractions were obtained from tissues using a previously published method [2]. Tissue samples (100 mg) were homogenized in 10 mL of isolation buffer (210 mM of sucrose, 2 mM of EGTA, 40 mM of NaCl, and 30 mM of HEPES, pH 7.4) using a tissue grind pestle. The homogenized samples were centrifuged for 10 min at 900× *g* and 4 °C. The supernatant was collected and centrifuged at 10,000× *g* for 10 min at 4 °C. The obtained pellet was resuspended in 500 µL of resuspension buffer (10 mM of Tris and 1 mM of EDTA, pH 7.4) and used for analysis. A Bradford dye binding protein assay (Bio-Rad, Hercules, CA, USA, Cat. No. 5000001) was performed to determine the protein content of mitochondrial extracts. The protein contents of mitochondrial extracts were used to normalize data from subsequent biochemical assays to account for potential differences in mitochondrial extraction efficiencies between normal and WB samples.

Citrate synthase activity was measured in extracted mitochondrial samples in duplicate using a commercially available kit (Cayman, Ann Arbor, MI, USA, Cat. No. 701040) that measured the production of SH-CoA. Reagent preparation and assay procedures followed manufacturer recommendations. First, an acetyl-CoA reagent (50 µL/well) was loaded on a 96-well plate. Then, mitochondrial extracts (30 µL/well) were loaded, followed by a 20 s centrifugation to remove all bubbles. Next, an oxaloacetate reagent (20 µL/well) was added to start the reaction. Absorbance at 412 nm was read immediately and then every 2 min for 20 min at 25 °C. Enzyme activity was calculated based on the formula provided by the manufacturer, normalized to the protein concentration of mitochondrial extracts, and expressed as µmols/min/mg protein.

Mitochondrial complex-II activity was measured in extracted mitochondrial samples in duplicate using a commercially available kit (MyBioSource, San Diego, CA, USA, Cat. No. MBS2570535) that measured enzyme activity by monitoring the reduction of ubiquinone to ubiquinol via succinate oxidation. Reagent preparation and assay procedures followed manufacturer recommendations. A master-mix provided by the manufacturer was prepared using the reagents (working solution), and the assay was performed on a 96-well plate. First, the assay working solution (190 µL/well) was loaded and incubated at 37 °C for 3 min. Next, mitochondrial extracts (20 µL/well) were added, and the plate was shaken for a few seconds before absorbance was read at 600 nm. The plate was then incubated for 30 min and read again at 600 nm to provide two-point data. Mitochondrial complex-II activity was calculated based on the formula provided by the manufacturer, normalized to the protein concentration of mitochondrial extracts, and expressed as U/g protein.

Total glutathione content was measured in extracted mitochondrial samples in duplicate using a commercially available kit (Cayman, Ann Arbor, MI, USA, Cat. No. 703002) based on an enzymatic recycling colorimetric method using the DTNB–glutathione reaction. Reagent preparation and assay procedures followed manufacturer recommendations. Mitochondrial extracts were first deproteinized. To assess glutathione concentration, deproteinized samples and glutathione standards (50 µL/well) were then loaded on a 96-well plate. Next, a freshly prepared assay cocktail (150 µL/well) was added to start the reaction. The plate was read at 412 nm after 25 min. Glutathione concentration (µM) was calculated based on the obtained absorbance and standard curve values and then normalized to the protein concentration of mitochondrial extracts.

Total ATP content was measured in tissue samples in duplicate using a commercially available colorimetric assay (Abcam, Waltham, MA, USA, Cat. No. ab83355) based on the phosphorylation of glycerol. Reagent preparation and assay procedures followed manufacturer recommendations. A total of 10 mg of tissue was homogenized with a Dounce homogenizer in 100 µL of assay buffer, and the homogenate was centrifuged to obtain a supernatant. To remove any enzymes that could potentially interfere with the assay, the samples were deproteinized using perchloric acid and neutralized with potassium hydroxide. The assay was conducted on a 96-well plate. Standards and samples (50 µL/well) were loaded on the plate, followed by the reaction mix (50 µL/well). The plate was incubated for 30 min before absorbance was read at 570 nm. The ATP concentration was calculated based on a standard curve, normalized to the protein concentration of the tissue as determined by a Bradford dye binding protein assay (Bio-Rad, Hercules, CA, USA, Cat. No. 5000001), and expressed as µmol/g protein.

### 2.4. Statistical Analysis

As the objective of this study was to investigate differences between normal broilers (no WB) and those exhibiting the WB condition, data for each type of tissue were treated as individual datasets and analyzed separately using Student’s *t*-test method (GraphPad Prism, Version 10.2.2) to compare normal versus WB samples. The assumptions of data normality and homogeneity of variance were evaluated and met. Differences between types of tissues were not analyzed. The results were considered significant at *p* < 0.05. The observed biochemical differences between the two groups (WB and normal) may not be independent of body weight. In the context of multiple biochemical comparisons, marginal *p*-values should be interpreted accordingly.

## 3. Results

### 3.1. Meat Quality Measurements

Broilers exhibiting WB were 10.4% heavier than normal broilers (Normal vs. WB, 3831 vs. 4228 g, *p* = 0.002). Breast meat pH values were greater in WB samples compared to normals at 24 h post-mortem (5.78 vs. 5.90, *p* = 0.03). Raw compression force values were 38.6% greater in WB compared to normal breast muscles (1.01 vs. 1.40 N, *p* = 0.04). Drip loss was greater in WB samples than normal samples at 12 h (3.83 vs. 7.62%, *p* = 0.0003) and 24 h (5.54 vs. 9.32%, *p* = 0.01) post-mortem. There were no significant meat color differences between the two groups on either the skin- or bone-side surfaces of the breast muscles (Table 1).

### 3.2. Biochemical and Enzyme Activity Assays

All data are presented in Table 2. All assays were conducted in mitochondrial extracts from tissues, except ATP. Citrate synthase activity was reduced in the muscle (1.30 vs. 0.99 µmols/min/mg protein, *p* = 0.02, 23.8% change), pancreas (1.25 vs. 1.03 µmols/min/mg protein, *p* = 0.03, 17.6% change), liver (0.44 vs. 0.35 µmols/min/mg protein, *p* = 0.03, 20.5% change) and duodenum (0.90 vs. 0.55 µmols/min/mg protein, *p* = 0.0006, 38.9% change) of broilers exhibiting WB compared to normal broilers (no WB). Mitochondrial complex-II activity was reduced in the muscle (18.04 vs. 7.61 U/g protein, *p* = 0.0002, 57.8% change), liver (79.25 vs. 48.57 U/g protein, *p* = 0.008, 38.7% change), and duodenum (68.95 vs. 35.21 U/g protein, *p* = 0.03, 48.9% change) of broilers exhibiting WB. Glutathione was reduced in the pancreas (30.50 vs. 28.25 µM, *p* = 0.04, 7.4% change), duodenum (55.10 vs. 39.94 µM, *p* = 0.01, 27.5% change), jejunum (67.36 vs. 45.33 µM, *p* = 0.0002, 32.7% change) and ileum (76.46 vs. 61.39 µM, *p* = 0.01, 19.7% change) of broilers exhibiting WB. Total tissue ATP was reduced in the muscle (2.44 vs. 2.22 µmol/g protein, *p* = 0.03, 9.0% change) and liver (2.32 vs. 1.90 µmol/g protein, *p* = 0.04, 18.1% change) tissue of broilers exhibiting WB.

## 4. Discussion

The objective of this study was to identify mitochondrial-related differences in multiple organs between broilers exhibiting severe WB and normal broilers (no WB). As expected, WB broilers were on average heavier than normal (no WB) broilers in this study, suggesting that broiler size differences may have contributed to observed changes with the WB condition. As it was important to obtain tissues as early post-mortem as possible, the initial assessment of the WB condition was conducted by the tactile and visual assessment of the broiler breast muscles immediately post-slaughter, and only those that fit strict criteria were selected. In this study, carcasses were designated as normal if their breast muscles did not exhibit any of the distinct phenotypic characteristics of the WB myopathy. Due to the inherent subjective nature of assessing the WB condition and to ensure a comparison of distinct phenotypes, carcasses that displayed mild or moderate degrees of WB myopathy were not utilized in this study. A post-deboning assessment of the breast filets and meat quality measurements were conducted to confirm the correct classification of WB and normal breast filets. In agreement with previous studies [13,14,15], breast filets with the WB myopathy exhibited greater pH, drip loss and compression force measurements at 24 h post-mortem compared to normal filets. Thus, the samples selected for analyses were deemed adequate to properly meet the objectives of this study.

In this study, citrate synthase activity and mitochondrial complex-II activity were measured in mitochondrial-enriched fractions from each tissue and served as indicators of mitochondrial function within these tissues. Across all tissue types, citrate synthase catalyzes the first step in the TCA cycle, and mitochondrial complex-II (also known as succinate dehydrogenase) links the TCA cycle to the electron transport chain. Citrate, produced in the mitochondrial matrix, acts as a carbon source for cytoplasmic biosynthesis and regulates energy pathways [16]. Citrate-derived metabolism feeds the TCA cycle to fuel mitochondrial complex-II, which contributes directly to ATP production [17]. This process also generates ROS within the tissue. Because these enzymes are closely linked to the TCA cycle, assessing both enzymes in this study provided a more comprehensive understanding of mitochondrial performance and potential energy production capacity in the tissues. Glutathione, which is the primary intracellular antioxidant buffer, was measured as an indicator of cellular oxidative stress as its levels fluctuate in response to ROS. During mitochondrial stress, glutathione levels decline, leading to increased ROS, which inhibits mitochondrial complex-II, resulting in reduced ATP synthesis and higher oxidative damage in tissues [18]. Several previous studies showed that the mitochondrial regulation of oxidative stress in the breast muscle of broilers may play a role in the development of the WB myopathy [9,12]. Data from the current study demonstrated that citrate synthase activity and mitochondrial complex-II activity were lower in several tissues from birds exhibiting the WB condition. These are the first data to demonstrate an association between the WB condition and mitochondrial-related alterations in tissues beyond just the breast muscle. However, as tissues in this study were collected after the WB characteristics were already present, further research is needed to determine whether systemic physiological factors potentially contribute to WB development. It is possible that the observed changes across multiple tissues may reflect the consequences of the myopathy itself or the underlying growth and metabolic characteristics associated with severe WB, rather than causal factors in WB onset. The impact of the degree of WB severity on the association with mitochondrial-related alterations in various tissues could not be determined from this study as breast meat with mild or moderate degrees of WB was not included.

In broilers, the duodenum section of the small intestine functions as the primary site for enzymatic digestion and the initial site of nutrient absorption. The reduced citrate synthase and mitochondrial complex-II activities in the mitochondrial extracts from the duodenum tissue of WB broilers in this study suggest potentially suboptimal gut functioning in affected broilers. These data are supported by previous reports showing that duodenum tissue from WB broilers exhibited reduced expression of genes related to mitochondrial function and gut barrier function, reduced mitochondrial complex-V activity, alterations in digestive enzyme activities, and altered tissue morphology [2,12]. The potential biological link between WB development in the breast muscle and alterations in the gut is not fully understood, as the observational nature of the current study precludes establishing a causal relationship based on the available data. Based on the body of literature describing the impact of gut microbiota and gut functioning on meat quality in livestock and poultry [19], as well as the gut–muscle axis as it relates to skeletal muscle wasting disorders [20,21], it is reasonable to hypothesize that gut functioning in rapidly growing broilers could play a role in WB development. Metabolic stress in the gut due to mitochondrial dysfunction could lead to inflammation, compromised barrier function, and altered microbiota interactions within the gut. Research linking gut microbiota and skeletal muscle health in other species has shown that the inflammatory signals produced in the gut can circulate systemically and affect peripheral tissues, including skeletal muscle [22,23,24,25]. When muscles experience metabolic stress and inflammation, they more often rely on anaerobic energy pathways, leading to a buildup of cellular byproducts which can alter how the muscle tissue functions, recovers, and grows [26,27,28]. Such changes could potentially contribute to the altered muscle composition in WB that leads to inferior breast meat quality characteristics. However, more direct mechanistic and longitudinal studies are needed to assess the specific relationship between gut functioning and WB development.

Research on liver disease has shown that reduced mitochondrial complex-II activity and ATP levels in the liver and pancreas can disrupt whole body energy balance [29]. With regard to meat, metabolic imbalances have been shown to indirectly impair meat quality by altering the supply of metabolic substrates needed for anaerobic glycolysis in muscle and affecting muscle energy status before and after slaughter [30,31,32]. It has been shown that WB disrupts muscle ATP status by inducing a severe energy crisis characterized by localized hypoxia, mitochondrial dysfunction, and depleted cellular ATP reserves [11]. While data from the current study suggest that mitochondrial function may be impaired in the liver tissue of broilers exhibiting the WB myopathy, further research is needed to determine whether this phenomenon is mechanistically connected to the energy metabolism issues associated with WB muscle.

In this study, fewer mitochondrial-related differences between normal and WB samples were observed in the ileum than the duodenum tissue. A possible reason that the citrate synthase activity of the ileum tissue was unaffected by the WB condition could be related to tissue differences in cellular energy demand [33]. There is a direct relationship between cellular energy demand and the activity of citrate synthase. Normally the ileum has a lower energy demand and metabolic activities compared to the upper segments of the small intestine (duodenum and jejunum), as well as other highly metabolic organs like the liver. High-demand organs like the liver rely on greater mitochondrial respiration to sustain metabolic activity [34]. Somewhat dissimilarly to citrate synthase activity, the mitochondrial complex-II activity in the ileum showed a numerically large (~48% reduction) but not quite statistically significant difference (*p* = 0.06) in WB samples due to the wide variation observed in the ileum measurements. Future research to directly measure tissue-specific energy demand, mitochondrial respiration, or metabolic flux could help explain tissue-to-tissue differences as they relate to WB.

The reduction in glutathione in mitochondria in this study indicates an increase in oxidative stress within tissues. Elevated ROS levels heighten oxidative stress, leading to greater damage to mitochondrial DNA and proteins. Such oxidative injury impairs the efficiency of the electron transport chain and ultimately reduces ATP production, weakening overall mitochondrial function [35]. Although we expected to see lower ATP levels in the ileum when the glutathione level was lower, the current data showed that there was no significant difference in ATP in the ileum between the two groups. It is worth mentioning that glutathione is not the only factor involved in ATP production. Cellular energy demand may also impact the level of ATP production [36]. The ileum generally requires less ATP compared to other sections of the intestine and liver due to its specialized function as the final segment of the small intestine, which primarily handles remaining absorption rather than high-intensity active transport and processing [37].

## 5. Conclusions

Previous research has shown mitochondrial alterations in the *Pectoralis major* of broilers exhibiting the WB myopathy when compared to unaffected broilers. In addition to the breast muscle where the WB condition is manifested, this study demonstrated that mitochondrial-related biochemical traits in the small intestine, liver, and pancreas are also affected in broilers exhibiting severe WB. As mitochondria help maintain ATP levels and regulate ROS production in cells, these findings suggest that the suboptimal functioning of organs and tissues beyond the breast muscle may be associated with the severe WB condition in broilers. These findings warrant further longitudinal and mechanistic studies to better understand the etiology of the WB myopathy and potential systemic physiological factors contributing to WB development.

## Figures and Tables

**Table 1:** Live body weight and breast meat quality measurements of normal broilers and broilers exhibiting the wooden breast condition.

	Normal	Wooden Breast	SEM ^1^	*p*-Value ^2^
Body weight (g)	3831	4228	83.35	0.002
pH, 24 h	5.78	5.90	0.05	0.03
Compression force (N)	1.01	1.40	0.18	0.04
Drip loss (%)				
12 h	3.83	7.62	0.91	0.0003
24 h	5.54	9.32	1.45	0.01
Color, skin side ^3^				
L*	59.35	61.08	1.33	0.56
a*	0.84	1.34	0.37	0.54
b*	13.09	14.18	0.73	0.46
Color, bone side				
L*	56.68	56.40	1.42	0.99
a*	1.71	1.43	1.04	0.88
b*	14.87	14.03	0.74	0.66

^1^ SEM: pooled standard error of means. *n* = 10/group. ^2^ *p*-value < 0.05 indicates statistical difference. ^3^ Lightness, L*; redness, a*; yellowness, b*.

**Table 2:** Biochemical and enzyme activity measurements in tissues from normal and wooden breast broilers.

	Normal	Wooden Breast	SEM ^1^	*p*-Value ^2^
Citrate synthase activity (µmols/min/mg protein)				
Muscle	1.30	0.99	0.12	0.02
Pancreas	1.25	1.03	0.09	0.03
Liver	0.44	0.35	0.03	0.03
Duodenum	0.90	0.55	0.08	0.0006
Jejunum	1.41	1.28	0.07	0.09
Ileum	1.21	1.22	0.03	0.97
Mitochondrial complex-II activity (U/g protein)				
Muscle	18.04	7.61	2.27	0.0002
Pancreas	46.00	19.93	12.42	0.05
Liver	79.25	48.57	10.40	0.008
Duodenum	68.95	35.21	14.85	0.03
Jejunum	29.00	22.32	6.29	0.30
Ileum	83.63	43.13	20.41	0.06
Glutathione (µM)				
Muscle	48.40	41.60	4.68	0.16
Pancreas	30.50	28.25	1.05	0.04
Liver	167.50	138.30	14.79	0.06
Duodenum	55.10	39.94	5.60	0.01
Jejunum	67.36	45.33	3.87	0.0002
Ileum	76.46	61.39	5.57	0.01
Adenosine triphosphate or ATP (µmol/g protein)				
Muscle	2.44	2.22	0.09	0.03
Pancreas	2.08	1.88	0.13	0.14
Liver	2.32	1.90	0.19	0.04
Duodenum	1.92	1.77	0.10	0.16
Jejunum	2.56	2.28	0.14	0.06
Ileum	1.69	1.63	0.08	0.50

^1^ SEM: pooled standard error of means. *n* = 10/group. ^2^ *p*-value < 0.05 indicates statistical difference.

## Data Availability

All data is presented within this text.

## Abbreviations

The following abbreviations are used in this manuscript:

WB	Wooden breast
ROS	Reactive oxygen species
ATP	Adenosine triphosphate
TCA cycle	Citric acid cycle
